# Genome-wide identification of long intergenic non-coding RNAs for *Ralstonia solanacearum* resistance in tomato (*Solanum lycopersicum*)

**DOI:** 10.3389/fpls.2022.981281

**Published:** 2022-09-16

**Authors:** Peina Cao, Chuang Zhan, Junliang Yin, Shuangjun Gong, Dongfang Ma, Yan Li

**Affiliations:** ^1^Engineering Research Center of Ecology and Agricultural Use of Wetland, Ministry of Education/College of Agriculture, Yangtze University, Jingzhou, China; ^2^Key Laboratory of Integrated Pest Management on Crop in Central China, Ministry of Agriculture/Hubei Province Key Laboratory for Control of Crop Diseases, Pest and Weeds/Institute of Plant Protection and Soil Science, Hubei Academy of Agricultural Sciences, Wuhan, China

**Keywords:** LincRNA, *Ralstonia solanacearum*, qRT-PCR, differentially expressed, target genes

## Abstract

There is growing evidences indicating that long intergenic ncRNAs (lincRNAs) play key roles in plant development and stress responses. To research tomato lincRNA functions during the interaction between tomato and *Ralstonia* s*olanacearum*, RNA-seq data of tomato plants inoculated with *R. solanacearum* was analyzed. In this study, 315 possible lincRNAs were identified from RNA-seq data. Then 23 differentially expressed lincRNAs between tomato plants inoculated with *R. solanacearum* and control were identified and a total of 171 possible target genes for these differentially expressed lincRNAs were predicted. Through GO and KEGG analysis, we found that lincRNA might be involved in jasmonic acid and ethylene signaling pathways to respond to tomato bacterial wilt infection. Furthermore, lincRNA may also be involved in regulating the expression of AGO protein. Subsequently, analysis of expression patterns between differentially expressed lincRNAs and adjacent mRNAs by qRT-PCR revealed that part of lincRNAs and their possible target genes exhibited positive correlation. Taken together, these results suggest that lincRNAs play potential roles in tomato against *R.* s*olanacearum* infection and will provide fundamental information about the lincRNA-based plant defense mechanisms.

## Introduction

As a piece of the human eating routine, tomato (*Solanum lycopersicum*) has been tamed for many years (Rambla et al., 2014). Currently, tomato has been the second most consumed vegetable in the world (after potato and before onion) (Bergougnoux, 2014). In 2020, the harvested area of tomato has reached 5,051,183 hectares and tomato production has reached 186,821,216 tons worldwide (data from FAO). However, tomato bacterial wilt, which was caused by *Ralstonia solanacearum*, is the serious disease during tomato production (Baichoo and Jaufeerally-Fakim, 2016).

*R. solanacearum* exists in the root surface, and then it could spread across the whole plant through xylem vessel. This bacterium could degrade the cell wall by releasing cellulase and pectinase, thereby inhibits nutrient and water transport in the tomato plant (Raza et al., 2016), eventually, causes the host plant to die and wilt within a few days (Vasse et al., 1995). *R. solanacearum* could be survived in soil for a long time. Wide host range and geographical distribution make it very hard to control (Murti et al., 2021). Host resistance is eco-friendly and low cost to control tomato bacterial wilt (Nguyen et al., 2021). Different susceptible genes (*S* genes) and resistance gene (*R* genes) can be identified by the RNA-seq approach (Gao and Bradeen, 2016).

Long intergenic ncRNAs (LincRNAs) are defined as transcribed non-coding RNAs, which are longer than 200 nucleotides (nt) and are located between two protein-coding genes without overlapping with annotated coding genes (Liu et al., 2012; Sanchita Trivedi and Asif, 2019). Previous studies have shown that lincRNAs are usually co-expressed with neighboring genes to play important regulatory roles in higher eukaryotes (Cabili et al., 2011; Wang J. X. et al., 2018). For example, lincRNAs in soybean are involved in stress response, signal transduction, and developmental processes (Golicz et al., 2017). In addition, lincRNAs are associated with epigenetic markers in rice (Wang et al., 2015). Some lincRNAs have been reported to dynamically regulate auxin to drive chromatin loop formation (Ariel et al., 2014), and also to be involved in the response to low-nutrient conditions in *Arabidopsis thaliana* (Fukuda et al., 2019). LincRNAs from populus (*Populus trichocarpa*) were found in response to drought stress (Shuai et al., 2014). LincRNAs were also found to be responsive to *Pectobacterium carotovorum* infection in *Solanum tuberosum* (Kwenda et al., 2016). Furthermore, lincRNA of wheat may play roles in response to stripe rust and powdery mildew infection (Zhang et al., 2016). These results indicated that lincRNAs play indispensable roles in regulating plant growth and stress responses. However, there are few studies on lincRNAs related to tomato disease resistance.

Many studies have demonstrated that lincRNAs play active roles in the resistance to various pathogens in different plants, there has yet to be few researches on them in tomato, especially in tomato-*R. solanacearum* interaction system. The expression patterns and functions of tomato lincRNAs during the interaction with *R. solanacearum* has not been studied extensively. In this work, we identified tomato lincRNAs in a genome-wide scale and investigated the responses of tomato to *R. solanacearum* infection. Furthermore, we predicted possible target genes of differentially expressed lincRNAs within 100 kb of chromosomal locations and profiled the expression patterns of several lincRNAs using qRT-PCR. All potential target genes were then functionally annotated to pick out genes of interest in response to *R. solanacearum* infection. Our findings will provide fundamental information about the lincRNA-based plant defense mechanisms, which is useful for future molecular breeding of pathogen-resistant plants.

## Materials and methods

### Transcriptome data collection

High-throughput RNA-seq data were downloaded from NCBI (accession number PRJNA787007). The samples were stems at the six-leaf stage from two tomato cultivars (resistance and susceptibility to bacterial wilt) seedlings. Each cultivar was inoculated with *R. solanacearum* as treatment group, and the control group was healthy plants without inoculation. Sample R (R0, R1) represents stem tissue taken from tomato cultivar with bacterial wilt resistance, and S (S0, S1) stands for the susceptible cultivar. There were four groups of samples with three biological replicates per group.

### RNA-seq reads mapping and transcriptome assembly

The SRA files were converted into fastq files using fasterq-dump (version 2.9.1). Fastqc (version 0.11.9) was used to detect the quality of the raw reads, and trim_galore software (version 0.6.7) was used to filter adapter content and low-quality reads. HISAT2 (version 2.0.1) was used to map the clean reads to the tomato reference genome which was downloaded from the Phytozome database.^1^ The format of output file was sam format. Samtools software (version 1.13) was used to converted and sorted sam files to bam files. Then the bam files are used as input files for StringTie (version 2.1.7) to get merged gtf files.

### Pipeline for long intergenic ncRNAs identification

The main program of lincRNA recognition are as follows: (1) GffCompare can be used to classify all the transcripts (the merged gtf files obtained above) in the input samples according to the reference transcript (Genome annotation file) (Pertea and Pertea, 2020). GffCompare software (version 0.11.2) was used to compare all the transcripts with the annotated information of the genome. Transcripts without the “u” character were filtered, and intergenic transcripts with the parameter “−r” of GffCompare were retained (Pertea et al., 2016); (2) Single exon transcripts less than 200 bp in length were removed; (3) Collect the location information of exons in the remaining transcript, extract the genome sequence of corresponding exons and fuse it into a complete transcript sequence; (4) CPC2, PLEK, and CNCI tools were used to evaluate the protein-coding potential of complete transcription sequences, transcripts with coding capacity are discarded (Kong et al., 2007); (5) Elimination of transcripts containing any known protein-coding domain. Transdecoder (version 5.5.0) was used to identify the open reading frame of the complete transcript sequence, and transcripts with ORF greater than 300 were removed; (6) Transeq program in EMBOSS (version 6.6.0.0) was used to translate the transcript sequence into six possible amino acid sequences, these protein sequences are required to be compared with known proteins in the Pfam database for homology, and those with high homology (*E*-value < 1e-5) are discarded (Finn et al., 2015); (7) The BLASTX program was used to analyze the homology of transcription-encoded proteins with known proteins in the NR database. When the *e*-value was less than 1e-5, the corresponding transcript was discarded (Pirooznia et al., 2008); (8) The expression level of the transcript was determined using parameters “−e” and “−B” in the StringTie (Pertea et al., 2016). Transcripts with read count > 0 in at least one sample are retained as potential lincRNAs.

### Characterization of tomato long intergenic ncRNAs

The exon number and transcript length of lincRNA and protein-coding genes were calculated using Excel software. GraphPad Prism 8 software was used to plot the violin plot of average gene expression.

### Differential expression analysis of long intergenic ncRNAs

Differential expression analysis was performed using the OmicShare tools- DESeq2.^2^ Expression abundance of lincRNA in different samples was obtained above. LincRNA whose expression levels in different treatment groups met both | log_2_(FC)| ≥ 1 and *P*-value < 0.05 was considered to be significantly differentially expressed.

### Prediction of differentially expressed long intergenic ncRNAs target genes

LincRNAs have been reported to regulate the expression of adjacent genes (Zou et al., 2018). In order to clarify the role of lincRNA of tomato after bacterial wilt infection, the potential cis-regulating target genes with differential expression of lincRNAs were predicted. Based on the tomato genome information, the mRNAs within 100 kb at 5′ upstream or 3′ downstream of each lincRNA are considered as potential cis-targets (Hou et al., 2017). Cytoscape software (version 3.9.1) was used to map the regulatory network.

### Functional annotation of long intergenic ncRNAs target genes

All predicted potential target genes were functionally annotated in order to single out genes for disease resistance. GO and KEGG functional enrichment was performed using OmicShare tools. LincRNAs related to *R. solanacearum* infection were selected, and the heatmaps were drawn based on the expression level of lincRNAs.^3^ Based on the annotated information of the genome, the function of adjacent target genes is obtained (Supplementary Table 6).

### Plant materials and *Ralstonia solanacearum* and *Ralstonia solanacearum* inoculation

Tomato (Shouhefenguan F1 generation) seeds were germinated on wet filter papers in an incubator at 28°C for 2 days in the dark (Zhu et al., 2020). Good seedlings were selected and transferred to individual 3-inch pots. The tomato plants were cultured under 28 ± 2°C for 16 h in light and 8 h in darkness. At the six-leaf stage, the seedlings were inoculated with *R. solanacearum* strain GMI1000 which was obtained from Professor Meixiang Zhang (Shaanxi Normal University). Single colonies of *R. solanacearum* from TTC medium were transferred into NB medium for 48 h at 28°C. After centrifugation at 4,000 rpm for 10 min, the bacteria were re-suspended with sterile distilled water to give an optical density of 1.0 at 600 nm (approximately 10^8^ cfu/mL). 50 mL of bacterial suspension was drenched over the soil surface for inoculation (Jaunet and Wang, 1999). The stem tissue was collected at 0, 2, 8, 12, 24, and 48 h after inoculation, then was frozen in liquid nitrogen, and was maintained in a cryogenic refrigerator. The samples at 0 h were used as control group.

### RNA extraction and cDNA synthesis

Total RNA from tomato stem tissues was extracted using the TRIzol reagent (Invitrogen, USA) and digested with DNaseI (TaKaRa, Beijing, China) to remove genome DNA. The RNA was reverse transcribed using RevertAid Reverse Transcriptase (Vazyme, Nanjing, China). The obtained cDNA was diluted to 100 ng/μL with enzyme-free water.

### Expression pattern validation by quantitative real-time PCR

To verify whether the putative lincRNAs have cis-regulatory functions on the predicted target genes, the expression patterns of several lincRNA were verified by qRT-PCR. Nine lincRNAs were selected for qRT-PCR. They are MSTRG. 4629.2-Solyc02g062230.1, MSTRG.16084.2-Solyc06g010060.1, MSTRG.14272.1-Solyc05g013220.2, MSTRG.14378.4-Solyc05g 014260.3, MSTRG.3272.2-Solyc01g104370.4, MSTRG.19303.2-Solyc07g039550.4, MSTRG.30350.1-Solyc12g014390.3, MSTR G.8436-Solyc03g058460.1, MSTRG.14616.2- Solyc05g018310.3. The internal reference gene is *Actin* (GenBank No. U60480.1). Primer 5.0 software and website^4^ were used to design the gene-specific primers. All the primer pairs used for PCR amplification were shown in Supplementary Table 6. The reactions were conducted in a 20 μL volume containing 10 μL 2 × *PerfectStart*™ Green qPCR SuperMix, 0.4 μL of each primer (10 μmol/mL), 7.2 μL double distilled water, and 2 μL of the template cDNA under the following conditions: 94°C for 30 s followed by 40 two-step cycles of 94°C for 5 s and 59°C for 30 s.

## Results

### Genome-wide identification of long intergenic ncRNAs in tomato

**FIGURE 1:**
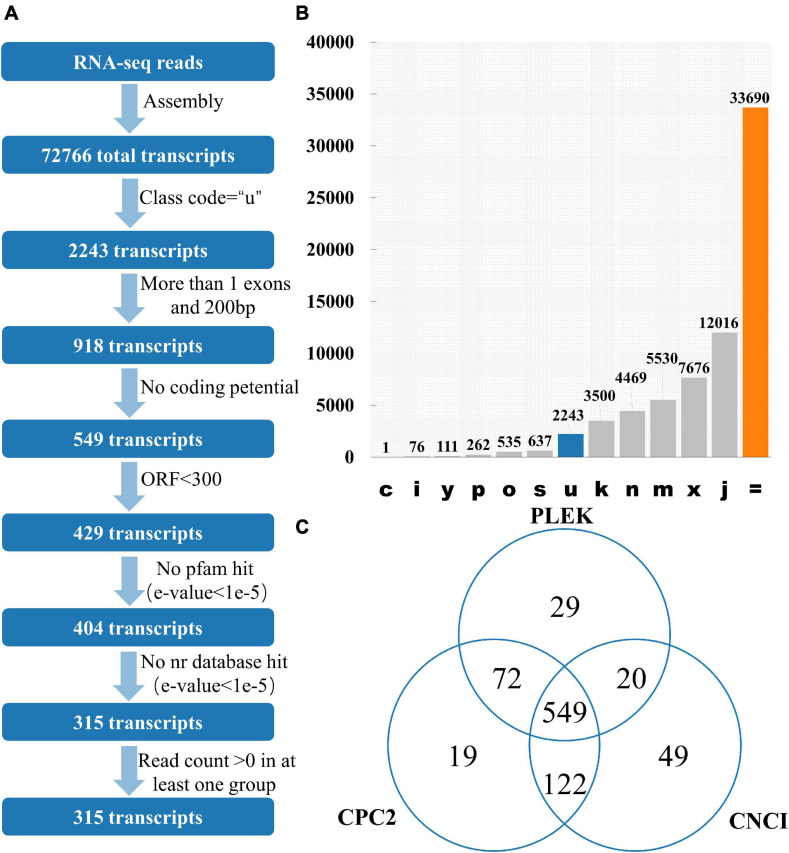
Identification of lincRNA from transcripts. **(A)** Pipeline for lincRNA Identification. **(B)** The number of each transcript. Class_code is used to indicate the position of the transcript relative to the reference genome. **(C)** Venn diagrams of transcripts without protein-coding ability evaluated by three software. Note: c represents the position of transcript relative to reference genome is contained in reference (intron compatible). i represents the position of transcript relative to reference genome is fully contained within a reference intron. y represents the position of transcript relative to reference genome is contains a reference within its intron(s). p represents the position of transcript relative to reference genome is possible polymerase run-on (no actual overlap). o represents the position of transcript relative to reference genome is other same strand overlap with reference exons. s represents the position of transcript relative to reference genome is intron match on the opposite strand (likely a mapping error). k represents the position of transcript relative to reference genome is containment of reference (reverse containment). n represents the position of transcript relative to reference genome is retained intron(s), not all introns matched/covered. m represents the position of transcript relative to reference genome is retained intron(s), all introns matched or retained. x represents the position of transcript relative to reference genome is exonic overlap on the opposite strand. j represents the position of transcript relative to reference genome is multi-exon with at least one junction match. = represents the position of transcript relative to reference genome is complete, exact match of intron chain. u represents the position of transcript relative to reference genome is none of the above (unknown, intergenetic). Class_code reference paper (Pertea and Pertea, 2020).

12 publicly available tomato transcriptomes (Supplementary Table 1) were downloaded. After quality control of transcriptome data such as removal of adapter and removal of low-quality reads, 97.43% of the reads were successfully aligned with the tomato reference genome using HISAT2 (Supplementary Table 1). Stringtie was used to perform the assembly process, and there are 72,766 transcripts for lincRNA identification. GffCompare software was used to classify all the transcripts and lincRNAs are usually selected with “class_code = u” (Pertea and Pertea, 2020; Figures 1A,B). Among the 72,766 transcripts, 2,243 of them had a class code of u, and 918 transcripts were remained after filtering out the single exon transcripts with a length less than 200 bp. Among them, CPC2 predicted results showed that 762 transcripts had no coding ability, PLEK prediction showed 670 transcripts had no coding capacity, and CNCI prediction indicated 740 transcripts had no coding capacity. The intersection of the three software results were 549 transcripts without coding capacity (Figure 1C). The length of the open reading frame (ORF) of the coding-capable mRNA typically greater than 300 nt. If the ORF of the RNA sequence is less than 300 nt, it is very unlikely to encode a protein. So, 429 transcripts with an ORF length less than 300 nt were finally retained. Transeq procedure was used to translate the transcript sequence into six possible protein sequences, which were compared with Pfam and Nr databases. The transcripts with high homology to the protein database were removed. Furthermore, lincRNAs with read count > 0 in at least one sample were considered to be expressed, and finally, 315 transcripts were obtained as putative tomato lincRNAs.

### Characterization of tomato long intergenic ncRNAs

**FIGURE 2:**
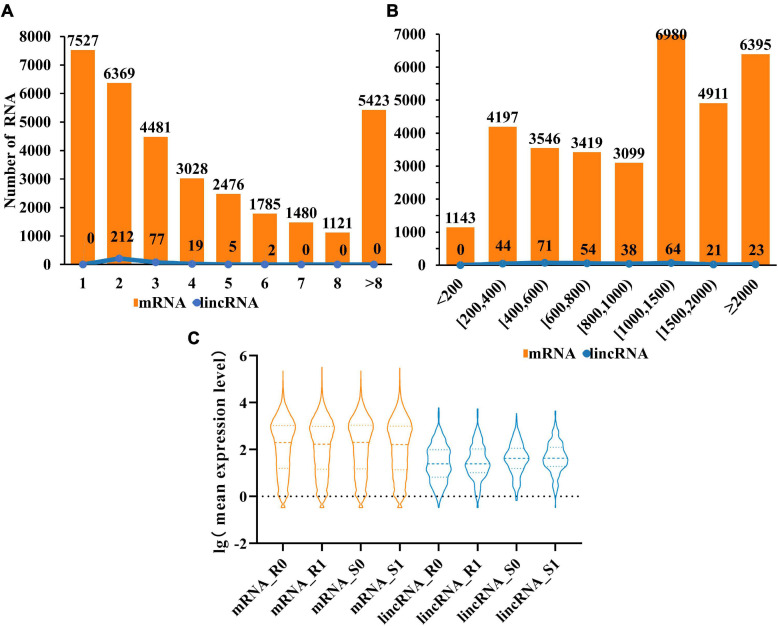
Characterization of tomato lincRNAs. **(A)** Compare the exon numbers of lincRNAs and protein-coding genes. **(B)** Compare the length of transcripts of lincRNAs and protein-coding genes. **(C)** The average expression levels of lincRNAs and protein-coding genes were compared (lincRNA in blue, mRNA in orange).

Compared with mRNAs, lincRNAs are smaller in length and have fewer exons has been reported (Zhu et al., 2015). To determine whether the lincRNAs in tomato have the above characteristics, we compared the transcript length and exon number of lincRNAs (315) and mRNAs (33,690) in tomato genome. The statistical data showed that the number of lincRNA and mRNA decreased gradually with the increase of the number of exons. It is noteworthy that the number of exons in the lincRNAs ranges from two to six. The result revealed that although the length of lincRNAs is greater than 200 nt, the length will not be particularly long (Figure 2A). Analysis of transcript length shows that the sequence length of 66% of lincRNA is between 200 and 1,000 nt, with 34% more than 1,000 nucleotides. However, for mRNAs, 54% were greater than 1,000 nt (Figure 2B). These results indicated that, unlike mRNAs, most of the tomato lincRNAs were shorter and had fewer exons. After analyzing the mean expression level of mRNAs and lincRNAs in different treatment groups, the results showed that the average expression levels of lincRNAs were significantly lower than mRNAs (Figure 2C). This may be the reason why lincRNA didn’t get noticed at first. In addition, the average expression level of lincRNAs also have obvious differences between different tomato cultivars and this difference is not obvious in mRNAs. The result indicated that lincRNAs are more susceptible to cultivars factors than mRNAs.

### Analysis of differentially expressed long intergenic ncRNAs and predicting the target genes

**FIGURE 3:**
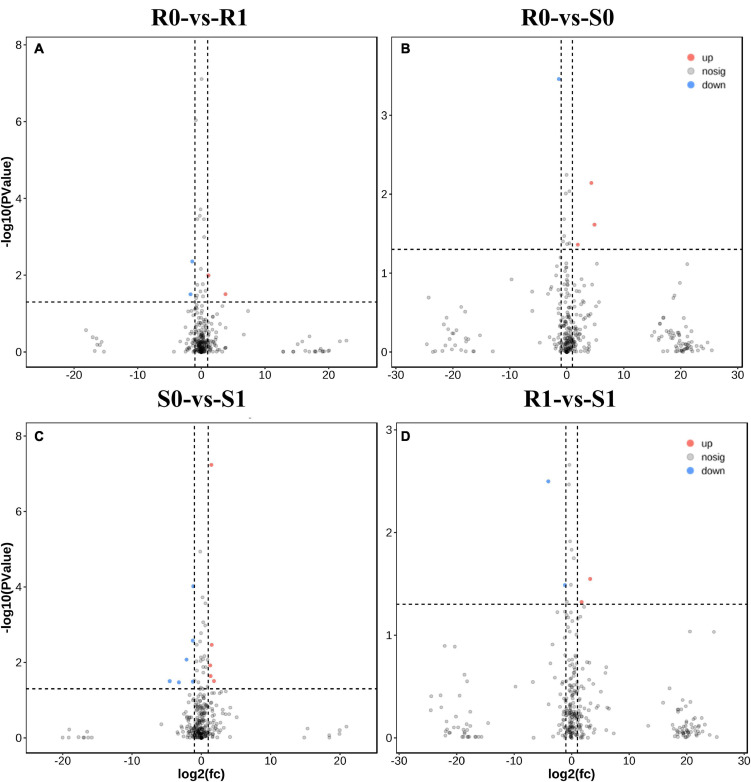
Differential expressions analysis of lincRNAs. **(A–D)** The volcano diagram of differential expression of lincRNA in different treatment groups was shown in sequence: R0 vs. R1, R0 vs. S0, S0 vs. S1, and R1 vs. S1.

**FIGURE 4:**
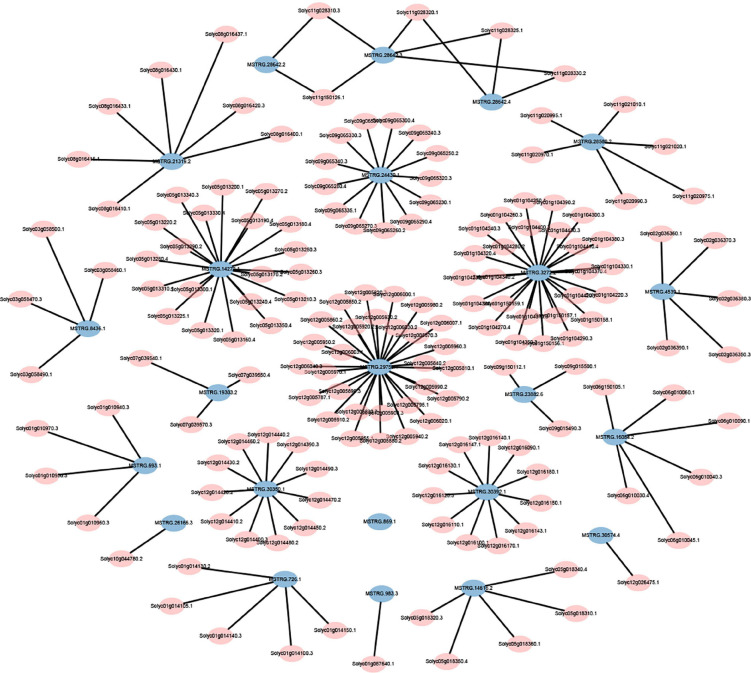
Distribution of adjacent functional genes with differential expression of lincRNAs (see Supplementary Table 3 for details).

**FIGURE 5:**
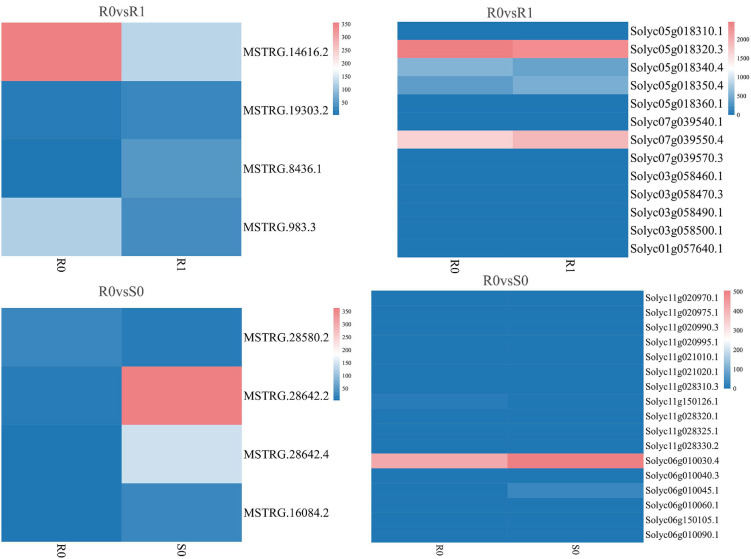
Expression of differentially expressed lincRNAs and adjacent mRNAs in R0 vs. R1, R0 vs. S0.

**FIGURE 6:**
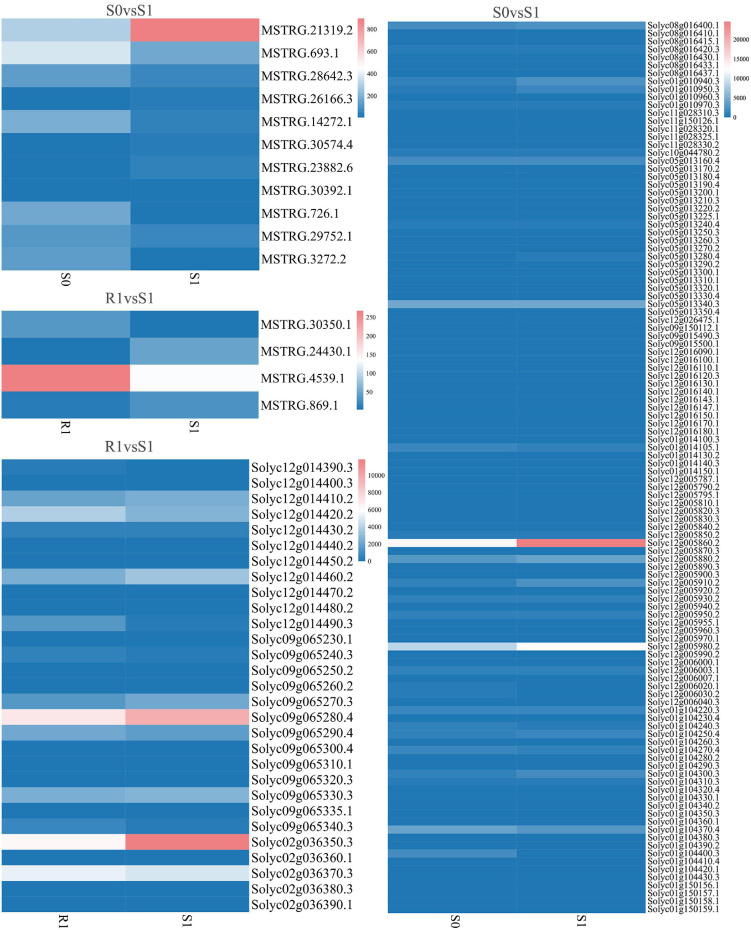
Expression of differentially expressed lincRNAs and adjacent mRNAs in S0 vs. S1, R1 vs. S1.

The expression levels of 315 lincRNAs in 12 samples were analyzed by differential expression analysis. The lincRNAs with at least twofold change in expression level and *P*-value < 0.05 were considered to be significantly differentially expressed. A total of 23 differentially expressed lincRNAs were selected from R0 vs. R1, S0 vs. S1, R0 vs. S0, and R1 vs. S1 in four comparison groups (Figure 3). It is worth noting that four differentially expressed lincRNAs were found between R0 and R1, while 11 differentially expressed lincRNAs were found between S0 and S1 (Supplementary Table 2). The results indicated that the lincRNAs response to *R. solanacearum* infection are different in different cultivars. Therefore, these lincRNAs with significantly differential expression obtained by pairwise comparison were collected together. After genome-wide comparison, 171 adjacent functional genes near 23 differentially expressed lincRNAs genes were screened (Figure 4 and Supplementary Table 3). The result revealed that a single lincRNA has multiple potential adjacent target Genes. In addition, heat maps of gene expression were plotted according to their expression levels (Figures 5, 6). The results of the heatmap hardly illustrate the connection between lincRNAs and mRNAs at expression levels.

### Functional annotations of target genes

**FIGURE 7:**
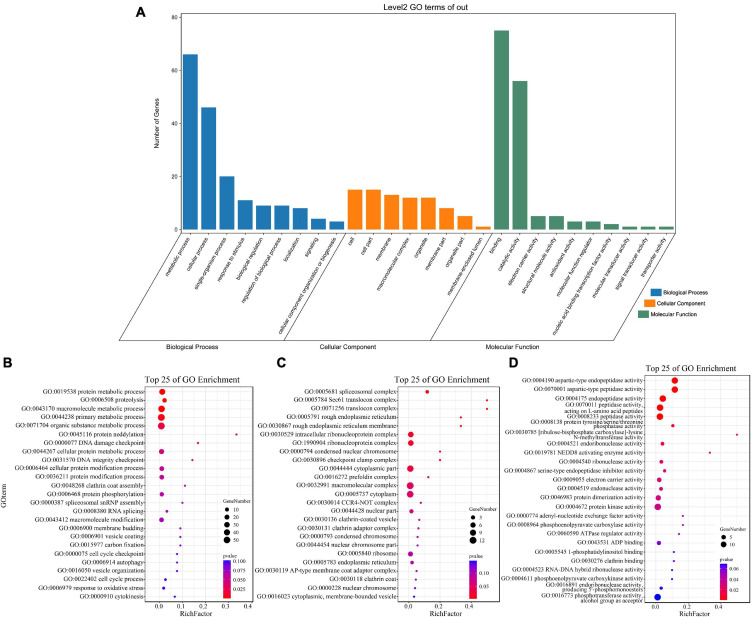
GO terms enrichment analysis of targets of 136 differentially expressed lincRNAs. **(A)** GO secondary classification statistics chart. The number of genes used for enrichment in each classification of GO was calculated. The abscissa represents the classification of GO three ontologies, namely, the secondary classification. The vertical axis represents the number of genes in each category. **(B–D)** Go enriched bubble map. Only enriched GO term (*P*-value < 0.05). The top 25 of biological process (BP), and the top 25 of molecular function (MF) and cellular component (CC) are shown. The circle size represented the gene number. Red to blue represents the low to high *P*-value.

**FIGURE 8:**
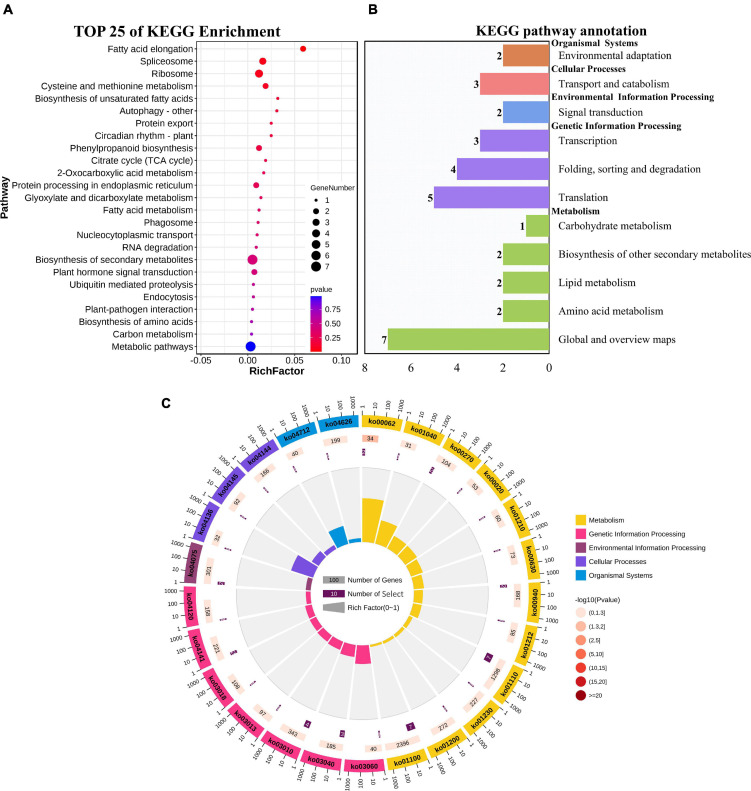
KEGG functional enrichment map of potential target genes of differentially expressed lincRNAs. **(A)** KEGG enrichment analysis of targets of 23 differentially expressed lincRNAs. The circle size represents the gene number. Red to blue represents the low to high *P*-value. **(B)** Statistical graph of number of class B gene annotations of Pathway. The names in bold black on the vertical axis are class A classification names, and the names in black are class B classification names. The horizontal axis represents the number of genes annotated to the corresponding B pathway. **(C)** Enrichment of circular graph. From the outside in, the first circle is the Pathway ID, Group colors correspond to different A class categories of KEGG, Outside the circle is the coordinate ruler of the number of genes; The bar length of the second circle corresponds to the number of background genes, the current pathway contains all genes, the color from dark to light corresponds to the *P*-value from small to large. The more genes, the longer the bar, and the smaller the *P*-value, the redder the color; the third circle corresponds to the number of target genes, the number of target genes contained in the current pathway; The polar histogram of the fourth circle is the rich factor, Represents the proportion of target gene in background genes.

GO enrichment and KEGG pathway analysis were used to annotate the functions of adjacent target genes of differentially expressed lincRNAs. Functional annotation analysis was performed on 171 predicted target genes. A total of nine, eight, and ten GO terms for biological process (BP), cellular component (CC), and molecular function (MF) were obtained, respectively (Figure 7A and Supplementary Table 4). For BP, most of genes were related to protein metabolic processes (GO: 0019538; 0019538) (Figure 7B). For CC and MF, the most significant enrichment is spliceosomal complex (GO: 0005681) and aspartic-type endopeptidase activity (GO: 0004190), respectively (Figures 7C,D). The KEGG pathway result showed that the 25 pathways were enriched (Figure 8A). Statistical chart of level B classification of each pathway showed that the target genes belonged to 5 grade A classifications and 11 grade B classifications. Most of the annotated genes play a role in the plant metabolic processes (Figure 8B). KEGG enrichment circle diagram showed that ko00062 Pathway was the most significant one (*P*-value < 0.05). Ko00062 Pathway is fatty acid elongation pathway, the target genes involved in this pathway are Solyc05g014150.4.1 and Solyc05g013220.2.1 (Figure 8C and Supplementary Table 5). Furthermore, the possible function of 171 adjacent target genes was obtained according to the annotated information in the tomato genome (Supplementary Table 3). The result revealed that Solyc01g010970.3, adjacent target gene of MSTRG.693.1, was predicted to be involved in argonaute family protein. The function of Solyc05g018320.3 (adjacent target gene of MSTRG.14616.2) may be related to novel interactor of JAZ. Jasmonic acid is known to have a defense-inducing effect in plants. Solyc02g036350.3 (adjacent target gene of MSTRG.4539.1) was predicted to be related to ethylene-forming enzyme. These findings suggest that lincRNA related to tomato bacterial wilt may respond to pathogen invasion by regulating jasmonic acid, ethylene pathways, and expression of AGO protein.

### The correlation between long intergenic ncRNAs and corresponding mRNAs expression patterns

**FIGURE 9:**
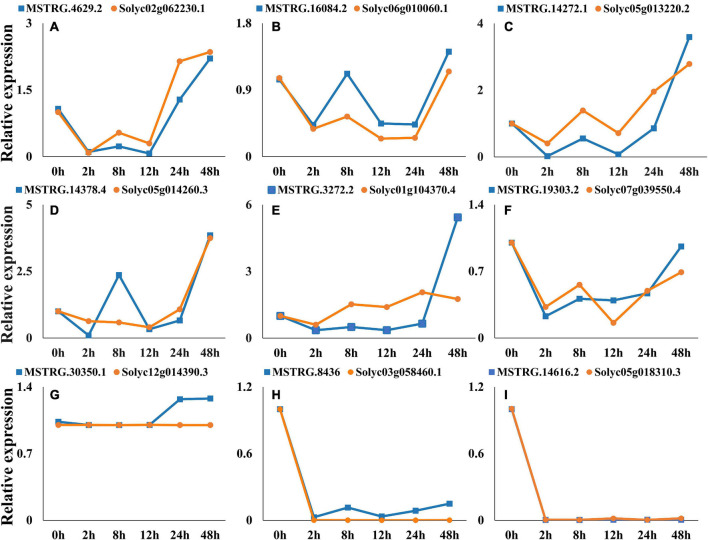
Expression pattern profiling of lincRNAs and mRNA pairs. The figure shows the expression pattern of lincRNAs. **(A)** MSTRG.4629.2-Solyc02g062230.1, **(B)** MSTRG.16084.2-Solyc06g010060.1, **(C)** MSTRG.14272.1-Solyc05g013220.2, **(D)** MSTRG.14378.4-Solyc05g014260.3, **(E)** MSTRG.3272.2-Solyc01g104370.4, **(F)** MSTRG.19303.2-Solyc07g039550.4, **(G)** MSTRG.30350.1-Solyc12g014390.3, **(H)** MSTRG.8436-Solyc03g058460.1, **(I)** MSTRG.14616.2- Solyc05g018310.3, interacting mRNAs at different times of infection.

Nine co-expressed lincRNAs and mRNAs were randomly selected, and the expression patterns were analyzed. A single lincRNA interacting with multiple mRNAs was detected. In this case, we selected the mRNA which is closest to lincRNA on the chromosomal for expression pattern analysis. The results show that expression patterns of randomly selected lincRNAs and corresponding mRNAs exhibited similar correlation in most of the infection time in tomato (Figures 9A–I). For instance, lincRNA “MSTRG.4629.2” interacting mRNA “Solyc02g062230.1” displayed positive correlation in expression trend at different times (Figure 9A). Moreover, a no-correlation could also be detected in several infection time, which might be either due to complex interaction network between lincRNA and mRNA or due to some unknown regions.

## Discussion

LincRNAs play key roles in regulating plant growth and in response to biotic and abiotic stresses. However, lincRNAs cannot be identified directly from genome and can be only identified from RNA seq data (Shumayla et al., 2017). Some features of lincRNAs such as the temporal, spatial, inducible, and various other specific expression patterns lead most of the studies in plants to be limited scale (Shumayla et al., 2017). In this work, we performed the identification of lincRNAs in tomato using the data from stems at the six-leaf stage from two tomato cultivars. After analyzing 12 RNA-seq data sets, and 315 possible lincRNAs were identified. These lincRNAs had fewer exons and shorter transcript length, which are markedly distinguished from mRNAs. Because of their low coding potential, their expression level is obviously lower than mRNAs. Ponjavic and Ponting (2007) found that about half of lincRNAs are transcribed near (<10 kb) to protein-coding genes, and these lincRNAs perhaps represent the best candidates for investigating the transcriptional regulation of neighboring genes. We expanded our search for neighboring genes to 100 kb of 5′ upstream and 3′ downstream of each lincRNA and 171 adjacent target genes was obtained. Furthermore, the expression patterns of lincRNAs interacting mRNAs were analyzed and part of the lincRNAs and corresponding mRNAs exhibited similar correlation in most of the infection time in tomato.

Although more and more studies have begun to focus on the identification of lincRNAs, the exploration of their functions still lags behind (Sanchita Trivedi and Asif, 2019). Because of the conservation of non-coding RNA sequences, they were initially thought to have no functions (Wang et al., 2004). In the regulation of gene expression, ncRNAs are involved in multiple mechanisms. The regulatory modality of small RNAs such as miRNAs and siRNAs has been widely reported (Pillai, 2005; Carthew and Sontheimer, 2009; Tiwari et al., 2014; Sanchita et al., 2018). These genes bind to their corresponding targets (mRNAs) to achieve gene silencing. Studies have found that lincRNAs also play critical roles in transcriptional and posttranscriptional regulation of gene expression in a wide variety of organisms (Fukuda et al., 2019). Later research found that long non-coding RNAs with potential functions in gene regulation are widespread throughout the transcriptome (Iyer et al., 2015). The non-coding region of the genome is largely composed of transposable elements, some of which are functionalized with lincRNAs (Zhao et al., 2021). How lincRNA regulates the expression of susceptibility or resistance genes in tomato to play what role needs further study. However, it has been reported that OsAGO2 negatively regulates OsHXK1 expression at the epigenetic level through DNA methylation of OsHXK1 promoter region, thus negatively regulating rice resistance to black stripe dwarf disease and some ARGONAUTE family members are required for RNAi-like phenomena (Hunter et al., 2003; Wang et al., 2021). The GO and KEGG analysis enlightened the probable function of potential target genes, however, the actual role needs to be established in future studies (Shumayla et al., 2017).

Compared to lincRNA, lncRNA is more deeply researched (lincRNA is a kind of lncRNA). Except for the length requirement, we found that lncRNAs lack a typical ORF, initiation codon, 3′ UTRs, and a termination codon. However, previous studies have shown that the formation of lncRNAs are only slightly different from protein-coding mRNAs. For example, most of the lncRNAs are transcribed by RNA polymerase II, very similar to mRNAs. Differences in lncRNAs transcription by RNA polymerase III were also found (Wu et al., 2012). Even lncRNAs is missing parts of its functional structure relative to mRNA, the lncRNAs still have all the properties like polyadenylation at 3′ end, 5′ capping and splicing (Quan et al., 2015). Similar research results indicate that lincRNAs also has methylation process, which is different from mRNAs lincRNAs had different DNA methylation profiles (Wang M. H. et al., 2018). And the researchers found that lincRNAs were obviously activated after CG DNA methylation (Zhao et al., 2021). Other studies have shown that the lincRNAs have two regulation of gene expression, cis and trans manner (Zhang et al., 2014; Quan et al., 2015). The various ways in which lincRNA functions are gradually being explored and discovered. Little by little, the mystery of lincRNA is being unraveled. Our research will focus on specific lincRNAs that play a key role in tomato infection by *R. solanacearum*. The potential lincRNAs of the tomato identified from our analysis have certain reference significance and these lincRNAs may be used for further functional genomics studies.

## Data availability statement

Publicly available datasets were analyzed in this study. This data can be found here: NCBI, PRJNA787007.

## Author contributions

JY and DM designed the article. PC and SG directed the data analysis and manuscript writing. CZ supervised the experiment. DM and YL confirmed the manuscript. All authors contributed to the article and agreed to submit version of the manuscript.

## Footnotes

1
https://phytozome-next.jgi.doe.gov/info/Slycopersicum_ITAG4_0

2
https://www.omicshare.com/tools

3
https://hiplot-academic.com/basic/heatmap?lang=zh_cn

4
https://biodb.swu.edu.cn/qprimerdb/
